# Study on Antibacterial and Quorum-Sensing Inhibition Activities of *Cinnamomum camphora* Leaf Essential Oil

**DOI:** 10.3390/molecules24203792

**Published:** 2019-10-21

**Authors:** Wenting Wang, Dongxiang Li, Xiaoqin Huang, Huixiang Yang, Ziwen Qiu, Liting Zou, Qin Liang, Yu Shi, Yingxiang Wu, Shaohua Wu, Chao Yang, Yongyu Li

**Affiliations:** 1 College of Horticulture, Fujian Agriculture and Forestry University, Fuzhou 350002, China; wangwtsci@163.com (W.W.); dongxiang_li@163.com (D.L.); 13305929270@163.com (X.H.); 13023832839@163.com (H.Y.); Qzw1996111@outlook.com (Z.Q.); Mizai_8zone@163.com (L.Z.); sychiyu@163.com (Y.S.); 2 Qingyuan Agricultural Science and Technology Extension Service Center, Qingyuan 511518, China; wuyingxing8501@163.com

**Keywords:** *Cinnamomum camphora* leaf essential oil, gas chromatography–mass spectrometry (GC-MS), antibacterial activity, *Chomobacterium violaceum* ATCC31532, quorum sensing (QS)

## Abstract

Many essential oils (EOs) regulate the quorum-sensing (QS) system of pathogens and inhibit the virulence expression. Interference with QS can potentially reduce bacterial multidrug resistance and aid the biological control of bacterial disease. In the present work, the antibacterial and anti-QS activities of *Cinnamomum camphora* leaf EO were investigated. A total of 23 chemical components with relative levels ≥0.11%, including a large number of terpene compounds, were identified in *C. camphora* leaf EO by gas chromatography–mass spectrometry (GC-MS). The principal component was linalool, followed by eucalyptol, with relative levels of 51.57% and 22.07%, respectively. The minimum inhibitory concentration (MIC) and antibacterial activity of *C. camphora* EO were examined, and *P. aeruginosa* and *E. coli* ATCC25922 showed the highest and lowest sensitivity to *C. camphora* EO, respectively. Tests of QS inhibitory activity revealed that *C. camphora* EO significantly decreased the production of violacein and biofilm biomass in *C. violaceum*, with the maximum inhibition rates of 63% and 77.64%, respectively, and inhibited the biofilm formation and swarming movement, independent of affecting the growth of *C. violaceum*. Addition of *C. camphora* EO also resulted in downregulation of the expression of the acyl-homoserine lactones (AHL) synthesis gene (*cviI*) and transcription regulator (*cviR*), and inhibited the expression of QS-regulated virulence genes, including *vioA*, *vioB, vioC*, *vioD*, *vioE*, *lasA*, *lasB*, *pilE3*, and *hmsHNFR*. Collectively, the prominent antibacterial activity and anti-QS activities clearly support that *C. camphora* EO acts as a potential antibacterial agent and QS inhibitor in the prevention of bacterial contamination.

## 1. Introduction

In recent years, antibiotics and antimicrobial agents have been widely used for the prevention and control of bacterial diseases. Traditional antimicrobial agents inhibit and kill bacteria by interfering with their biochemical metabolism as well as by affecting their structure and functions [1]. Bacteria rapidly mutate under selective pressure, which results in rapid increases in drug-resistant bacteria and leads to many difficulties in the prevention and treatment of bacterial diseases. Therefore, the discovery of different novel therapies to treat or decrease cases of bacterial infections is urgently required. Inhibition of quorum sensing (QS) may be a suitable solution. Bacterial quorum sensing is a regulatory mechanism in bacterial colony communications whereby bacteria produce and secrete specific signal molecules and perceive the changes in the bacterial quantity in their surrounding environment through the changes in the concentration of the signal molecule (autoinducer, AI). QS endows a large number of individual bacteria with the ability to coexpress certain genes under specific conditions. Bacterial activities, including the release of pathogenic factors, the formation of biofilms, fluorescence, the formation of spores, the production of antibiotics, and DNA transcription, can be regulated by QS [2]. Studies have shown that quorum sensing (QS) is capable of regulating the activities of protein, fat, pectin, chitosan, and other decomposing enzymes related to pathogenic bacteria, and different types of QS signal molecules have also been detected in diseased tissue [3]. To date, a few antimicrobial agents with broad-spectrum antimicrobial activity have been shown to have QS inhibitory effects at sub minimal inhibitory concentration (sub-MIC) [4,5]. Many QS inhibitors (QSIs) of plant origin can inhibit the QS activities and reduce the pathogenicity of pathogens without inhibiting bacterial growth and these QSIs have the advantage of both not inducing drug resistance and being capable of preventing and controlling bacterial contamination [6]. Therefore, plant natural products could be the useful biological control agents targeted at bacterial QS networks. *Chromobacterium violaceum* and *Pseudomonas aeruginosa* PAO1 are well-known bioindicators used to identify substances that can block the QS mechanism [7,8].

Studies have shown that plant essential oils (EOs) can be used as QSIs to control bacterial diseases due to their broad and prominent antibacterial properties [9]. In addition, plant-derived EOs are the safe alternative to chemical antimicrobial agents. Husain et al. [10] found that *Mentha piperita* EO interfered with the production of AHL signal molecules and the biofilm formation in gram-negative bacteria and showed significant QSI activity. A study by Vattem et al. [11] hypothesized that the mechanism by which oregano essential oil reduces violacein may be associated with its principal component, carvacrol, which may inhibit the expression of AHL synthesis genes. Eugenol, the principal component of *Cinnamomum verum* EO, in combination with antibiotics, synergistically inhibited the QS activity of *Escherichia coli* [12]. The curcumin in *Curcuma longa* inhibited the formation of bacterial biofilms and QS-mediated physiological behaviors [13]. Trans-cinnamaldehyde inhibited AHL signal molecule synthesis by interacting with the substrates of the signal molecule synthase and thus inhibited the QS activity of *P. aeruginosa* [14].

*Cinnamomum camphora*, a species of evergreen trees in the *Lauraceae* family, is a primary tree species in the subtropical broad-leaved forest and is a commonly used aromatic tree species for afforestation in China. The roots, bark, leaf, and fruits of *C. camphora* can be used for medicinal purposes and are rich in EOs with antibacterial, anti-inflammatory, and insect-repellent properties [15]. Notably, *C. camphora* EO is the primary raw material for camphor and borneol in the medical industry. The antimicrobial activity of *C. camphora* has been studied recently, but its anti-QS ability has never been described, thus we identified the components of *C. camphora* EO and demonstrated antibacterial activity and QS inhibitory activity. We provided theoretical and experimental support for the development of *C. camphora* EO into a potential biological control agent.

## 2. Results

### 2.1. Analysis of the Components in *C. camphora* EO

**Figure 1:**
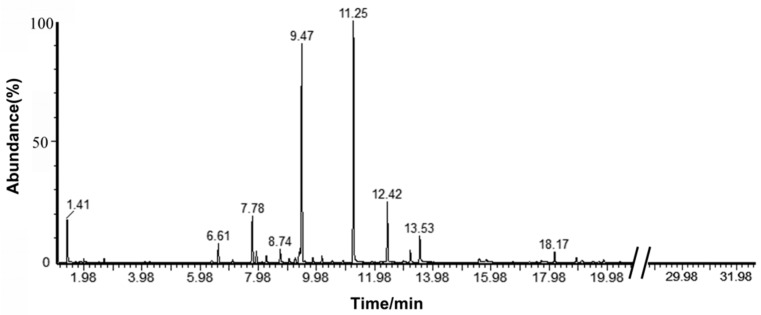
The GC-MS total ion chromatogram of *C. camphora* EO.

The yield of EO extracted from *C. camphora* leaf by steam distillation was 1.4%. A total ion flow chromatogram of the *C. camphora* EO analyzed by gas chromatography–mass spectrometry (GC-MS) is shown in Figure 1. Correlations of the peak area normalization results with the mass spectrometry database were performed to qualitatively and quantitatively analyze the components of the EO. Table 1 shows that 23 components with relative levels ≥0.11% were identified from the *C. camphora* EO, accounting for 97.55% of the total content. The *C. camphora* EO primarily contained linalool, eucalyptol, sabinene, α-terpineol, caryophyllene, nerolidol, α-pinene, camphor, and β-pinene, which accounted for more than 90% of the total content. The principal component was linalool (51.57%), followed by eucalyptol (22.07%), which indicated that the *C. camphora* in this study belonged to the linalool type.

### 2.2. Determination of Antimicrobial Activity and MIC of *C. camphora* EO

The antimicrobial activity of the *C. camphora* EO is shown in Table 2. *C. camphora* EO demonstrated significant inhibitory effects against *S. aureus* ATCC25933, *E. coli* ATCC25922, *P. aeruginosa*, and *C. violaceum* ATCC31532. The inhibitory effect was enhanced with increasing concentrations of *C. camphora* EO and *P. aeruginosa* exhibited a greater inhibitory effect than *S. aureus* ATCC25933, *E. coli* ATCC25922, and *C. violaceum* ATCC31532 at the same concentration.

The minimum inhibitory concentration (MIC) is defined as the minimum sample concentration with no obvious bacterial growth. The MIC of *C. camphora* EO was assessed for all test pathogens using doubling dilution method with concentration varying from 80‰ to 2.5‰. The MIC of *C. camphora* EO was 2.5‰ for *P. aeruginosa*, 5‰ for *S. aureus* ATCC25933 and *C. violaceum* ATCC31532, and 10‰ for *E. coli* ATCC25922.

The results of antimicrobial activity and MIC indicated that *P. aeruginosa* and *E. coli* ATCC25922 showed the highest and lowest sensitivity to *C. camphora* EO, respectively.

### 2.3. The Effect of *C. camphora* EO on Violacein of *C. violaceum* CV026

**Figure 2:**
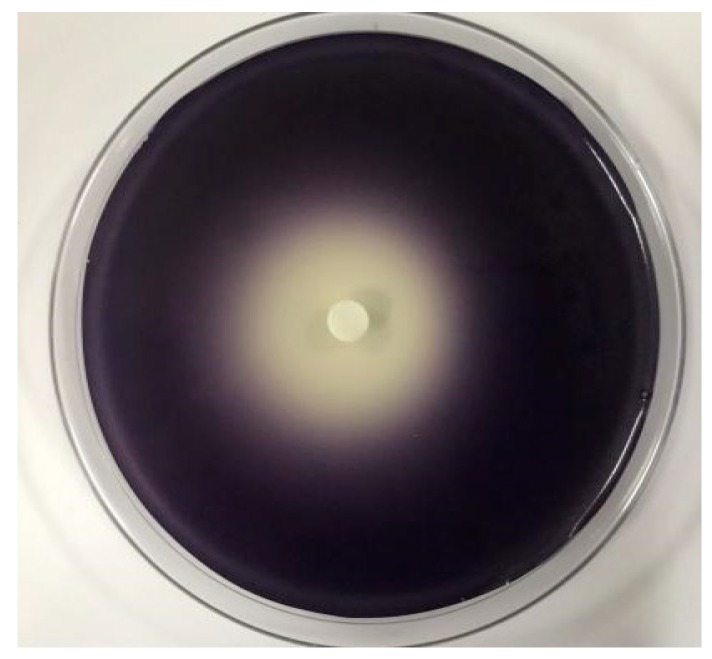
QSI effect of *C. camphora* EO on biosensor CV026.

The zone diameters of non-violacein on agar plate and the inhibition extent of violacein production in CV026 by *C. camphora* EO were both observed in Figure 2, which indicated that *C. camphora* EO had a good inhibitory effect on the QS-mediated violacein production of CV026. Thus, further experiments in the present study were performed at sub-MIC concentrations (2.5‰, 1.25‰, 0.625‰, and 0.3125‰) of *C. camphora* EO against *C. violaceum* ATCC31532.

### 2.4. Growth Curve Analysis

**Figure 3:**
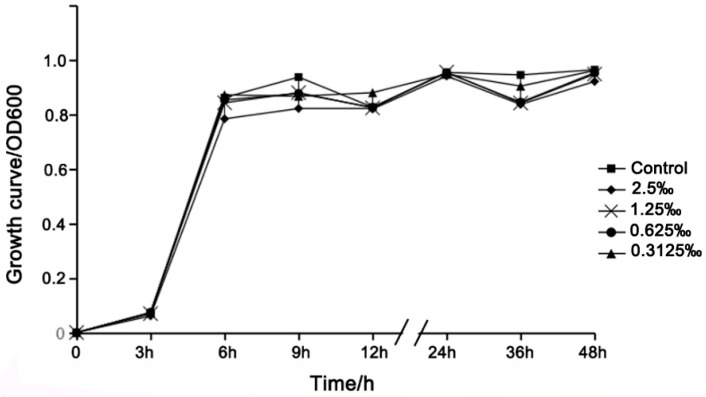
The effect of different concentrations of *C. camphora* EO on the growth of *C. violaceum*.

In the early stage of treatment (0–3 h), the *C. violaceum* in the treatment groups and control group was in the lag phase. At 3–6 h, the *C. violaceum* was in the logarithmic phase, in which the treatment groups contained different concentrations of *C. camphora* EO. During the later stage of treatment (≥6 h), the *C. violaceum* was in stationary phase in both the control group and the treatment groups, and there was no significant distinction among the groups (Figure 3). These results indicate that the *C. camphora* EO had no inhibitory effect on the growth of *C. violaceum* under the tested concentrations. We therefore assessed the specific effect of *C. camphora* EO on QS in *C. violaceum*.

### 2.5. Violacein Detection in *C. violaceum*

**Figure 4:**
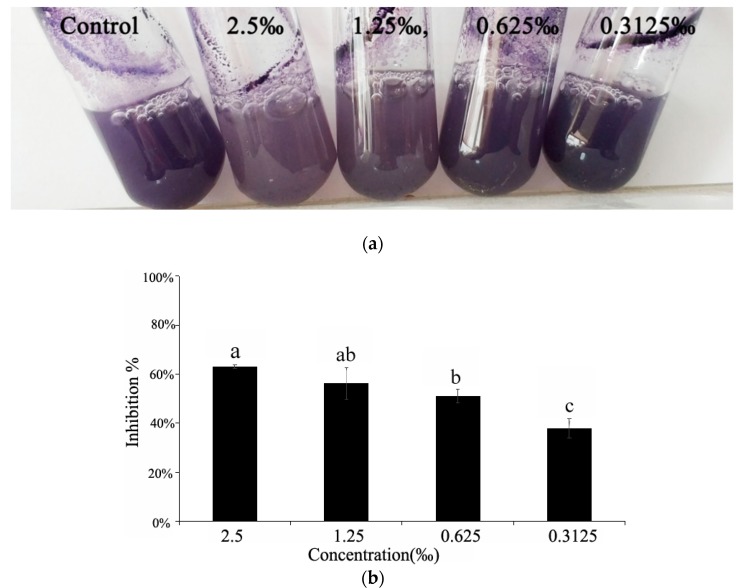
The effect of different concentrations of *C. camphora* EO on the production of violacein. (**a**) Effect of *C. camphora* EO on violacein production in *C. violaceum*. (**b**) Quantitative analysis of violacein inhibition in *C. violaceum* by *C. camphora* EO. Mean values of triplicate independent experiments and SD are shown. Bars indicate standard errors and different letters (**a**–**c**) above the bars represent significant differences (*p* < 0.05).

The synthesis of violacein is regulated by the QS system, and violacein is usually used as a simple and intuitive indicator for screening QSIs [16]. In our assay, we observed that *C. camphora* EO showed a notable and concentration-dependent inhibition of violacein production, which was dependent on QS regulation (Figure 4a). The results of purification and quantitative analysis of the violacein in the culture supernatant demonstrated the inhibitory effect of *C. camphora* EO on violacein production, and the maximum inhibition (63%) in *C. violaceum* occurred upon treatment with *C. camphora* EO at 2.5‰. (Figure 4b).

### 2.6. Effect of *C. camphora* EO on Biofilm Development

**Figure 5:**
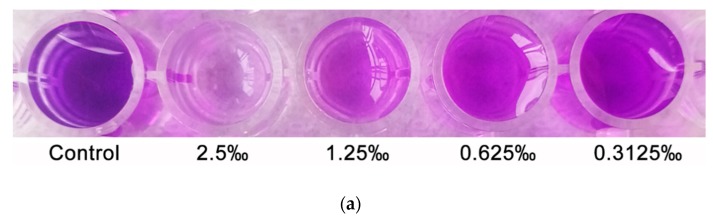

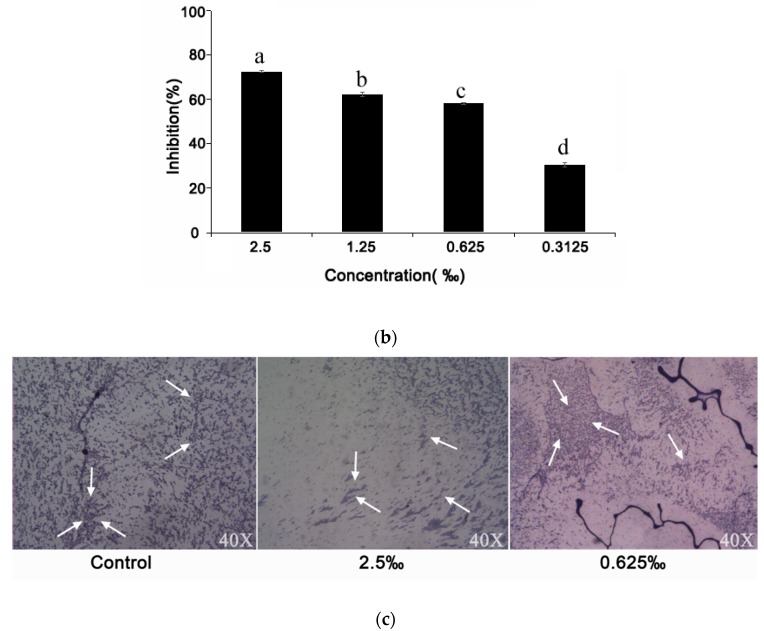
The effect of different concentrations of *C. camphora* EO on the biofilm. (**a**) Dyeing effect of crystal violet on biofilm of *C. violaceum* treated with different concentrations of *C. camphora* EO. (**b**) Quantitative analysis of the inhibition of biofilm biomass in *C. violaceum* by *C. camphora* EO. (**c**) Effect of *C. camphora* EO on biofilm formation. Mean values of triplicate independent experiments and SD are shown. Bars indicate standard errors and different letters (**a**–**d**) above the bars represent significant differences (*p* < 0.05).

Biofilms enhance bacterial tolerance to antibiotics, environmental pressure, and attacks by the host immune system. The formation of bacterial biofilms is regulated by the QS system [17]. The antibiofilm activity of *C. camphora* EO could be observed by biofilm staining with crystal violet (CV) (Figure 5a). The quantitative results exhibited an obvious concentration-dependent reduction in the biofilm biomass of *C. violaceum*. The *C. camphora* EO at sub-MIC (2.5‰, 1.25‰, 0.625‰, and 0.3125‰) decreased the biofilm biomass by 77.64%, 69.21%, 64.03%, and 26.83%, respectively (Figure 5b). In addition, we found that *C. camphora* EO influenced biofilm formation. Compared with the control group, the biofilms of *C. violaceum* in the EO-treated groups were distributed loosely and did not aggregate (Figure 5c).

### 2.7. Swarming Motility

**Figure 6:**
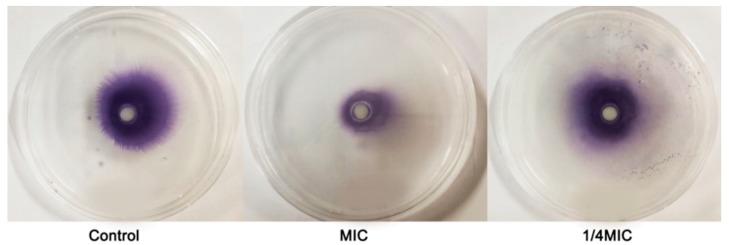
Inhibition of *C. camphora* EO on *C. violaceum*.

Swarming movement refers to the use of flagella to migrate from the inoculation point to the surrounding area. This colony-level activity depends on bioactive substances on the surface of the medium in a colony manner [18]. Swarming plays an important role in the preliminary stage of QS-regulated bacterial biofilm formation. Our results showed that *C. camphora* EO inhibited the swarming motility behavior of C. violaceum, and clear inhibition was observed upon treatment with sub-MICs (2.5‰ and 0.625‰) (Figure 6).

### 2.8. Expression of the QS-Related Gene in Response to *C. camphora* EO

**Figure 7:**
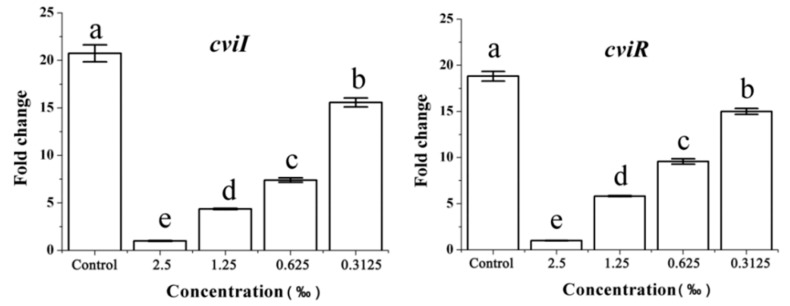
Effect of *C. camphora* EO on the expression of *cviI* and *cviR*. Quantitative RT-qPCR of the *cviI* and *cviR* genes. Expression of the housekeeping gene, *rpoD*, was used as the internal control for each sample. Bars indicate standard errors and different letters (**a**–**e**) above the bars represent significant differences (*p* < 0.05).

We further assessed the effects of *C. camphora* EO on the expression of QS genes in strain *C. violaceum* by RT-qPCR. As expected, the expression of the *cviI* and *cviR* genes decreased with an increase of *C. camphora* EO concentration and displayed the concentration-dependent change, independent of a direct effect on growth rate (Figure 7).

**Figure 8:**
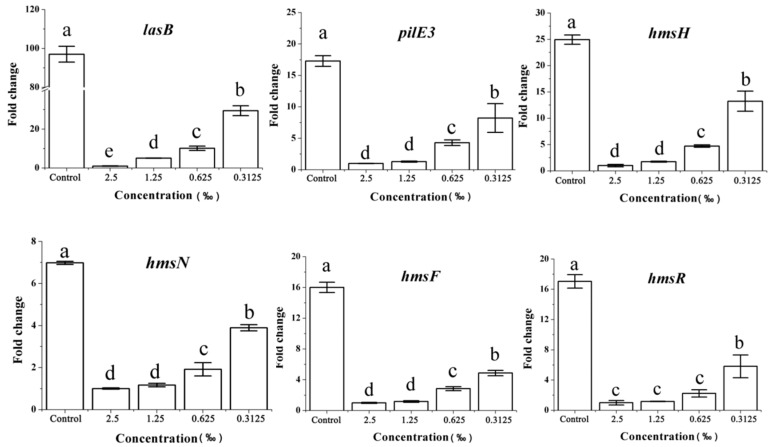
Effect of *C. camphora* EO on the expression of genes regulated by *LuxI-LuxR* system. Quantitative RT-qPCR of the *vioA, vioB, vioC, vioD, vioE, lasA, lasB*, and *pilE3* genes. Expression of the housekeeping gene, *rpoD*, was used as the internal control for each sample. Bars indicate standard errors and different letters (**a**–**e**) above the bars represent significant differences (*p* < 0.05).

Violacein synthesis and biofilm formation are important virulence factors in *C. violaceum*. Accordingly, we also evaluated the effect of *C. camphora* EO on the expression levels of genes that are directly involved in violacein synthesis and biofilm formation, including *vioABCDE* and *hmsHNFR*. These genes are downstream of the QS cascade and are strictly controlled by the QS system [19]. The relative expression of the gene clusters *vioA, vioB, vioC, vioD, vioE, hmsH, hmsN, hmsF*, and *hmsR* were clearly reduced by *C. camphora* EO relative to the control group (Figure 8). Furthermore, the effect of *C. camphora* EO on the relative expression of genes *lasA-B* and *pilE3*, encoding bacterial elastase and pilus, respectively, was assessed. *lasA* and *lasB* genes may be involved in limiting the host response, activating inappropriate host responses [20]. *PilE3*, which encodes a type IV pilus protein, and these fimbriae are involved in the enhancement of QS over aggregation and biofilm formation [21]. These genes have been associated with virulence in *C. violaceum*, but their expression has not been reported to be directly associated with QS. Consistent with the clear effect observed on QS and QS-regulated genes, the expression of *lasA-B* and *pilE3* was affected by exposure to the *C. camphora* EO (Figure 8).

## 3. Discussion

Resistance to antibiotics and chemical drugs is one of the greatest public health problems. This problem is a natural consequence of the adaption of infectious pathogens to antimicrobials used in several areas, including in medicine, food animal production, crop production and disinfectants in farms, hospitals, and households [22]. To find novel antimicrobial agents with new modes of action, plants have been explored as sources for the identification of new and effective antimicrobials. Plant EOs have shown clear inhibitory effects against fungi, bacteria, and viruses. Eos are natural, environmentally protective, safe, nontoxic, and widely present, thus indicating the great value of the application and utilization of Eos [23]. Our study also showed that *C. camphora* EO had marked antibacterial activity against the pathogens, including *S. aureus* ATCC25933, *E. coli* ATCC25922, *P. aeruginosa*, and *C. violaceum* ATCC31532. The multiple components of EOs often play a synergistic role in antibacterial activities, thus plant EOs exhibit not single-target but rather multitarget synergistic mechanisms [24].

The infection ability of bacteria can be effectively reduced by inhibiting the QS system, which will not threaten the growth of bacteria or prevent drug resistance [25]. Plant EOs exhibit great potential against pathogen contamination due to their prominent antibiofilm and anti-QS properties. Studies have shown that bacteria treated with sub-MIC concentrations of EO or their chemical individual constituents (ICs) were highly insensitive to bactericides and physical treatments, indicating that EOs or their ICs could reduce the incidence of bacterial drug resistance [26,27]. While planktonic bacteria are already resistant to many antimicrobials, in biofilms this resistance can increase several times. Biofilms may be formed on a variety of surfaces including living tissues, indwelling medical devices, and contact lenses [28]. Biofilm formation by pathogens is one of the most notable aspects of their pathogenicity and resistance, and QS plays a vital role in biofilm formation, thus interference with the QS system might be a preferable and convenient method to block its pathogenicity [25]. The inhibition of pathogen biofilms by *C. camphora* EO can effectively reduce the incidence of bacterial drug resistance, which makes this EO a suitable agent to combat pathogens.

QSIs interfered with the QS system primarily through the following three pathways: (1) the signaling molecule synthesis protein LuxI is affected such that the signal molecules, known as autoinducers (AIs), cannot be produced normally; (2) by degradation of the signaling molecules (AIs) to interfere with the expression of QS-related genes, even when the cell reaches the threshold population density; and (3) by prevention of the binding of the signaling molecule (AHL) to receptor protein LuxR, resulting in the inactivation of the transcriptional functions [29]. Plant phenolic aromatic compounds, including carvacrol and eugenol, directly interact with the signal molecule synthase and receptor proteins of *Pectobacterium aroidearum* PC1 and *P. carotovorum* subsp. *brasiliense* Pcb1692, thereby inhibiting the expression of QS-related genes, the biofilm formation, and synthesis of plant cell wall degradation enzymes (PCWDE) [30]. Our results showed that the *C. camphora* EO (sub-MIC) disturbed the swarming movement of *C. violaceum*, reduced the production of violacein and biofilm biomass, and inhibited biofilm formation. Inhibition of the QS major components (*CviI/CviR*) and virulence genes (*vioA, vioB, vioC, vioD, vioE, lasA, lasB pilE3*, and *hmsHNFR*) exposed to *C. camphora* EO was further reflected in lower virulence activities (violacein production, biofilm formation, and swarming movement). Our studies showed that *C. camphora* EO can reduce the bacterial pathogenicity by quenching bacterial QS instead of killing bacteria, providing a new pathway for the biological control of bacterial contamination.

Studies have shown that linalool and eucalyptol, as the main components in *C. camphora* EO, are capable of inhibiting bacterial biofilm formation [31,32]. We inferred that linalool (51.57%) and eucalyptol (22.07%) might be the important factors that interfered with the QS activity of *C. violaceum*. Linalool and eucalyptol may interfere synergistically with the AHL synthase and/or receptor proteins LuxR of *C. violacem* and regulate the expression of QS genes, thus affecting related physiological activities. The specific regulatory mechanisms still require further study. In the future, we plan to isolate bacterial QSIs from the *C. camphora* EO using the QS activity tracing method to clarify the regulatory mechanism and provide new methods for the biological control of pathogen contamination.

## 4. Materials and Methods

### 4.1. Materials

*Cinnamomum camphora* leaf was harvested on the campus of Fujian Agriculture and Forestry University (Fujian, China); a voucher specimen of the plant (*Cinnamomum camphora* (L.) Presl. officinal bar code No 00189531) was confirmed and deposited in the Institute of Botany, Chinese Academy of Sciences. The *C. camphora* leaf (*Cinnamomum camphora* (L.) Presl) in this study was identified by Professor Fangying Li in the College of Art and Landscape Architecture of Fujian Agriculture and Forestry University (Fujian, China).

Bacterial strains, medium, growth conditions: *Chromobacterium. violaceum* ATCC31532, *Chromobactrium violaceum* CV026, *Staphylococcus aureus* ATCC25933, *Escherichia coli* ATCC25922, and *Pseudomonas aeruginosa* were stored in the College of Horticulture in Fujian Agriculture and Forestry University (Fujian, China), and inoculated in LB broth and grown under the conditions of 30 °C, 150 rpm for 12 h.

*N*-hexanoyl-l-homoserine lactone (C6-HSL) was purchased from Sigma-Aldrich (Germany). n-alkanes (C8~C20) standard solution was purchased in Fluka.

### 4.2. Equipment

The gas chromatography–mass spectrometry technology (GC-MS) (GC, Clarus^®^680; MS, SQ8T; Perkin Elmer (USA), Shanghai branch (China)), ultraviolet spectrophotometer (U-290, Hitachi Company, Tokyo, Japan), Biobiology incubator (BPMJ-150F, Yiheng Company, Shanghai, China), ELISA (iMark, Bio-Rad Company, Hercules, CA, USA).

#### Extraction of *C. camphora* EO by Steaming

50 g of *C. camphora* leaf was placed in a round-bottom flask supplemented with distilled water at a 1:10 ratio. The EO was distilled in the essential oil extractor. The *C. camphora* EO was collected and the yield was calculated, and the calculation formula was shown in Equation (1). The experiment was repeated three times.

Yield of essential oil (%) = (weight of essential oil/weight of leaf) × 100%(1)

### 4.3. Determination of Components of *C. camphora* EO by GC-MS

The *C. camphora* EO was subjected to a GC-MS analysis using a GC (Clarus^®^680) equipped with a mass selective detector (SQ8T) in the electronic ionization (EI) mode and Turbomass Ver 6.1.0 software. The method was performed by using the following procedure with slight modification [33,34]. Sample injection was performed in split mode (20:1) into a DB-5MS capillary column, 30 m × 25 mm × 0.25 µm film thickness coated with 5% Ph Me Siloxane. Helium was used as the carrier gas at 1 mL min^−1^. The GC injector temperature was set at 200 °C. The oven temperature program was optimized to hold at 60 °C for 5 min, then heat to 150 °C at a rate of 3 °C/min (maintain for 5 min), and finally increase by 50 °C/min up to 250 °C (maintained for 2 min). The transfer line temperature was adjusted to 200 °C. Mass spectrometry conditions were as follows: electron ionization source set to 70 eV, MS transmission line to 250 °C, MS source to 230 °C. The mass spectrometer was run in full-scan mode (*m*/*z* 45–550). The essential oil production was analyzed by peak area.

The essential compounds were identified on the basis of a comparison of their retention index (RI) relative to n-alkanes (C8~C20), standard substance, as well as published data and EI mass spectra from the literature. Compounds were further compared and their MS data authenticated to the National Institute of Standards and Technology mass spectral library (NIST). The relative mass fraction of the EO was calculated using the peak area normalization method. The formula for retention index (RI) [35,36] is as follows in Equation (2):

RI = 100Z + 100[RT(x) − RT(z)]/[RT(Z + 1) − RT(z)](2)

where Z = the number of C in the smaller alkane; RT(x) = the retention time of the unknown compound; RT(z) = the retention time of the smaller alkane; RT(z + 1) = the retention time of the larger alkane.

### 4.4. The Determination of Antimicrobial Activity and MIC of *C. camphora* EO

The test strains, *S.aureus* ATCC25933, *E.coli* ATCC25922, *P. aeruginosa*, and *C. violaceum* ATCC31532 were cultured in LB broth at 150 rpm, 30 °C for 12 h. The *C. camphora* EO was serially diluted to 50%, 25%, and 12.5% with methanol. The plate perforation method was performed by using the following procedure with some modification [37]. Briefly, 1% overnight cultured test bacterium was added to the heated LB medium (containing 2% agar) at a temperature of 50 °C. After blending, the mixing was poured into the petri dishes quickly. Then, the solidified LB medium was punched with 6 mm holes every 120° in the petri dishes. A 25 µL volume of *C. camphora* EO at different concentrations (100%, 50%, 25%, and 12.5%) was added to the holes. The culture plates were incubated at 30 °C. The antimicrobial activity was measured by the antimicrobial diameters. Methanol and kanamycin (250 ug/mL) were used as negative control and positive control, respectively.

Doubling dilution method was applicable to test the minimum inhibitory concentrations (MIC) of *C. camphora* EO. The method was performed by using the following procedure with some modification [38]. Briefly, 1% of *C. violaceum* ATCC31532, *S. aureus* ATCC25933, *E. coli* ATCC25922, *P. aeruginosa* were added to LB medium (150 μL) supplemented with twofold serially diluted *C. camphora* EO (150 μL) to attain final concentration ranging from 2.5‰ to 80‰ of an equal final volume in 1.5 mL microcentrifuge tube, which was incubated for 24 h (30 °C, 150 rpm). The OD600 was measured.

### 4.5. Quorum-Sensing Inhibition (QSI) Assays of *C. camphora* EO

Quorum-sensing inhibition (QSI) assay was performed according to the procedure described by Zhang et al. [39] with a few modifications. An overnight culture of CV026 (1%) was spread on LB plates (20 mL), and then C6-HSL solution (2 µL, 50 mg/mL) was added to the plates. The filter paper (6 mm in diameter) was then placed into the center of the plate. Next, the *C. camphora* EO (15 µL) was added to the filter paper, and the plates were incubated for 24 h at 30 °C. QSI was assessed from the formation of a ring of inhibition extent of violacein production around the filter paper.

### 4.6. Growth Curve Analysis

The 1% *C. violaceum* was incubated in a 250 mL Erlenmeyer flask containing 20 mL of LB broth supplemented with different concentrations of *C. camphora* EO (sub-MIC, 1.2 mL). The mixes were cultured at 30 °C, under 150 rpm in a rotatory shaker. Bacterial growth was determined by UV-visible spectrophotometry at OD600 during a 3–48 h period.

### 4.7. Violacein Detection in *C. violaceum*

Violacein synthesis of *C. violaceum* strain ATCC31532 was regulated by the quorum sensing [40]. An overnight culture of 1% *C. violaceum* was added into a glass tube containing 5 mL LB broth supplemented with various concentrations (sub-MIC, 300 μL) of *C. camphora* EO. The mixture was incubated at 30 °C for 24 h. The specific methods were followed as described [41]. Briefly, 1 mL of the above solution was collected and centrifuged at 4 °C for 20 min. The supernatant was removed and 1 mL dimethyl sulfoxide (DMSO) was added to the centrifuge tube. After vortexing, the violacein was dissolved in DMSO at room temperature and centrifuged for 3 min to remove the bacterium. Violacein production was measured by UV-visible spectrophotometry at OD595, and the calculation formula was shown in Equation (3). Each assay was performed in triplicate.

Violacein inhibition rate(%) = (OD595_(Contol group)_ − OD595_(Treatment group)_)/OD595_(Contol group)_ × 100% (3)

### 4.8. Effect of *C. camphora* EO on Biofilm Development

The effect of *C. camphora* EO on biofilms was determined by quantifying the biofilm biomass using a microtiter dish with crystal violet (CV) and microtiter dish assay [42]. An overnight culture of 1% *C. violaceum* was added into a 96-well dish containing 200 μL of LB broth supplemented with various concentrations of *C. camphora* EO (sub-MIC, 6% *v*/*v*). The mixture was incubated at 30 °C for 24 h. After incubation, the unattached cells and media components were removed with distilled water. Then, 250 μL of a 0.1% solution of CV was added to each well of the microtiter plate and incubated at room temperature for 10–15 min. Finally, the 30% acetic acid was added for further determination. The absorbance at 600 nm was read in a microplate reader and recorded as the absorbance of CV dye bound to the bacterial biofilm. Each assay was performed in eight replicates.

To identify the ability of *C. camphora* EO to disrupt the biofilm, a biofilm disrupting assay was performed by following the revised method [43]. Briefly, the test pathogens treated with different concentrations of *C. camphora* EO (sub-MIC) were incubated in a 24-well plate with cover glasses 1 × 1 cm for 24 h and then were observed under a light microscope.

### 4.9. Swarming Motility

We examined the effect of *C. camphora* EO on the swarming motility in *C. violaceum*, following a previously described method [13]. Simply, 5 μL overnight cultured bacterium was incubated in 6 mm filter paper at the center of the swarming motility medium containing 1% tryptone, 0.5% NaCl, 0.5% agar, and 0.5% of D-glucose. Swarming motility was measured by the migration distance.

### 4.10. Gene Expression Analysis

1% *C. violaceum* (OD600, 0.9) was incubated with 20 mL LB containing a range of concentrations sub-MIC of *C. camphora* EO (1.2 mL). Cultures were grown for 24 h, the cells were harvested by centrifugation (12,000× *g*, 2 min), and supernatant was discarded. Total RNA was extracted using an RNAprep Pure Cell/Bacteria Kit (Code No. DP430, TIANGEN, China), according to the manufacturer’s guidelines. The RNA was used for reverse transcription, using Transcript One-Step gDNA Removal and cDNA Synthesis SuperMix (Transgen, China). The oligonucleotide primers were designed and are listed in Table 3. Quantitative RT-qPCR was performed using Real-Time PCR Master Mix (SYBR Green) (Transgen, China) under the following conditions: two steps of 30 s at 94 °C and 40 cycles of 94 °C for 5 s, 60 °C for 30 s. The calculated cycle threshold of each gene was normalized to the CT for *rpoD* amplified from the corresponding sample. The RT-qPCR was performed in Light cycler96 (Roche company, France). Fold changes in gene expression were calculated according to the 2^−ΔΔCT^ method.

### 4.11. Statistics Analysis

All experiments were performed at least in triplicates and all data were analyzed by SPSS 19.0 software and presented as mean values. Differences with *p* < 0.05 were considered statistically significant.

## 5. Conclusions

Twenty-three components with a relative mass fraction of ≥0.11% were identified in *C. camphora* EO, and the principal component was linalool (51.57%), which indicated that the species belongs to the linalool type.

The *C. camphora* EO had a significant inhibitory effect against *S. aureus* ATCC25933, *E. coli* ATCC25922, *P. aeruginosa*, and *C. violaceum* ATCC31532. In addition, the MICs of *C. camphora* EO were 2.5‰ for *P. aeruginosa*, 5‰ for *S. aureus* ATCC25933 and *C. violaceum* ATCC31532, and 10‰ for *E. coli* ATCC25922. Thus *P. aeruginosa* and *E. coli* ATCC25922 showed the highest and least sensitivity to *C. camphora* EO, respectively.

The *C. camphora* EO also interfered with the QS phenotype behaviors without inhibiting its growth, primarily as follows: the violacein and biofilm were reduced with the maximum inhibition rates of 63% and 77.64%, respectively. Biofilm formation and the swarming movement of *C. violaceum* were inhibited upon treatment with *C. camphora* EO. Furthermore, the expression of QS-related genes decreased significantly in the presence of *C. camphora* EO at sub-MICs.

Quorum-sensing quenching could be an alternative strategy to combat bacterial infections as it lowers the development of multidrug resistance. The anti-QS activity of *C. camphora* EO lays a foundation for developing camphor EO into a new biological pesticide targeting the bacterial QS system. However, the further study of the interaction mechanism between EO and bacteria should be analyzed in detail.

## Figures and Tables

**Table 1:** Chemical composition of *C. camphora* EO.

NO.	Compound Name	Molecular Formula	Molecular Weight	Relative Content	Retention Time/min	Retention Index
1	Linalool	C_10_H_18_O	154.25	51.57%	11.25	1021
2	Cineole	C_10_H_18_O	154.25	22.07%	9.47	981
3	Sabenene	C_10_H_16_	136.23	5.38%	12.42	1046
4	α-Terpineol	C_10_H_18_O	154.25	3.81%	7.78	939
5	Caryophyllene	C_15_H_24_	204.35	2.77%	13.54	1069
6	Nerolidol	C_15_H_26_O	222.37	1.97%	6.61	911
7	1R-α-Pinene	C_10_H_16_	136.23	1.68%	8.74	963
8	camphor	C_10_H_16_O	152.23	1.42%	7.92	943
9	β-Pinene	C_10_H_16_	136.23	1.12%	9.43	980
10	Terpineol	C_10_H_18_O	154.25	0.89%	13.20	1062
11	Methyleugenol	C_11_H_14_O_2_	178.23	0.83%	18.17	1167
12	a-Humulene	C_15_H_24_	204.35	0.76%	9.39	986
13	Myrcene	C_10_H_16_	136.23	0.56%	8.26	951
14	Caryophyllene oxide	C_15_H_24_O	220.35	0.45%	10.18	998
15	g-Terpinene	C_10_H_16_	136.23	0.40%	15.81	1117
16	α-Phellandrene	C_10_H_16_	136.23	0.35%	18.91	1182
17	Limonene	C_10_H_16_	136.23	0.32%	17.71	1157
18	Elixene	C_15_H_24_	204.35	0.26%	9.86	990
19	*cis*-4-Thujanol	C_10_H_18_O	154.25	0.24%	9.25	975
20	Terpinolene	C_10_H_16_	136.23	0.22%	19.09	1186
21	Germacrene D	C_15_H_24_	204.35	0.19%	17.71	1157
22	α-Selinene	C_15_H_24_	204.35	0.16%	6.38	905
23	2-isopropyltoluene	C_10_H_14_	134.22	0.11%	19.89	1203

**Table 2:** Antibacterial effect of *C. camphora* EO.

Bacteria	Concentration/Antimicrobial Diameters (mm)						MIC
	100%	50%	25%	12.5%	Methanol	Kanamycin (250 µg/mL)	
*Escherichia coli* ATCC25922	11.94 ± 0.235 ^a^^h^	11.81 ± 0.607 ^a^^h^	10.89 ± 0.545 ^b^^g^	8.44 ± 0.144 ^c^^g^	6.00 ± 0.00	15.72 ± 0.15	10‰
*Staphylococcus aureus* ATCC25933	14.35 ± 0.489 ^a^^f^	13.46 ± 0.364 ^b^^f^	11.74 ± 0.432 ^c^^f^	10.22 ± 0.020 ^d^^f^	6.00 ± 0.00	20.78 ± 1.81	5‰
*Pseudomonas aeruginosa*	16.61 ± 0.44 ^a^^e^	15.19 ± 0.60 ^b^^e^	13.59 ± 0.41 ^c^^e^	11.71 ± 1.17 ^de^	6.00 ± 0.00	21.02 ± 0.41	2.5‰
*Chromobacterium violaceum* ATCC31532	13.93 ± 1.09 ^a^^g^	12.37 ± 0.57 ^b^^g^	11.89 ± 0.74 ^bc^^f^	10.52 ± 0.17 ^c^^f^	6.00 ± 0.00	19.89 ± 2.33	5‰

Note: Different letters (a–d) within the same row represent significant difference at the different concentration (*p* < 0.05). Different letters (e–h) within the same line represent significant difference at the different concentration (*p* < 0.05).

**Table 3:** PCR Primers for Fluorescence Real-Time Quantitative PCR.

Gene	Forward (5′-3′)	Reverse (5′-3′)
*cviI*	GAAACCGTCCTCGCATAAGG	ACAAGGTGGACTGGTACTGG
*cviR*	CCCAGCAATATGCCGCTATC	CATTGAGCTTGCGGATCACA
*vioA*	AAGAGCATGGCAAGGAATC	CTGGTTGGCGTCGTTATC
*vioB*	CTGGGCGTAATTGGGAATGG	CAAATACCTGGCCCATGTCG
*vioC*	GAACAAGTACGCCAACCT	GGAAGAAAGTCTGCTGGAA
*vioD*	GCCGCAACAAGTACATCT	GAAGGTGCTCATCGTGTC
*vioE*	ATAGGCCACCTTCTGCTTCC	GGCTACTGCTGGTTCGACTA
*hmsH*	CGCCGTATGTCTTCAGTT	CGCAGCCTATCGTAGATG
*hmsF*	GGCTGCTGATTCTCTGTTA	GTATAGACGCTGCGGTAG
*hmsN*	AAACCACACGCACCAGAAC	TGCATGAGCATGAAGACGAC
*lasA*	AGCCAGCCTTACGATTCCAT	GAGGAATAGCCGTTGTCGTG
*lasB*	AGAACGCCTTGTTGTACACG	GCAAGAACGACTTCCTGGTC
*pilE3*	TACGCTGGTCGAGTTGATGA	GCGAATAGCACTGCTCCATC
*rpoD* *(actin)*	TCGGACATCAGCAAGGTT	GTGAAGGACAGCCAACAG
